# Supplementary figures and images for: Detection of pathogenic bacteria in ticks from Isiolo and Kwale counties of Kenya using metagenomics
Source: PLoS One. 2024 Apr 30;19(4):e0296597. doi: 10.1371/journal.pone.0296597 (PMC11060535; doi:10.1371/journal.pone.0296597)

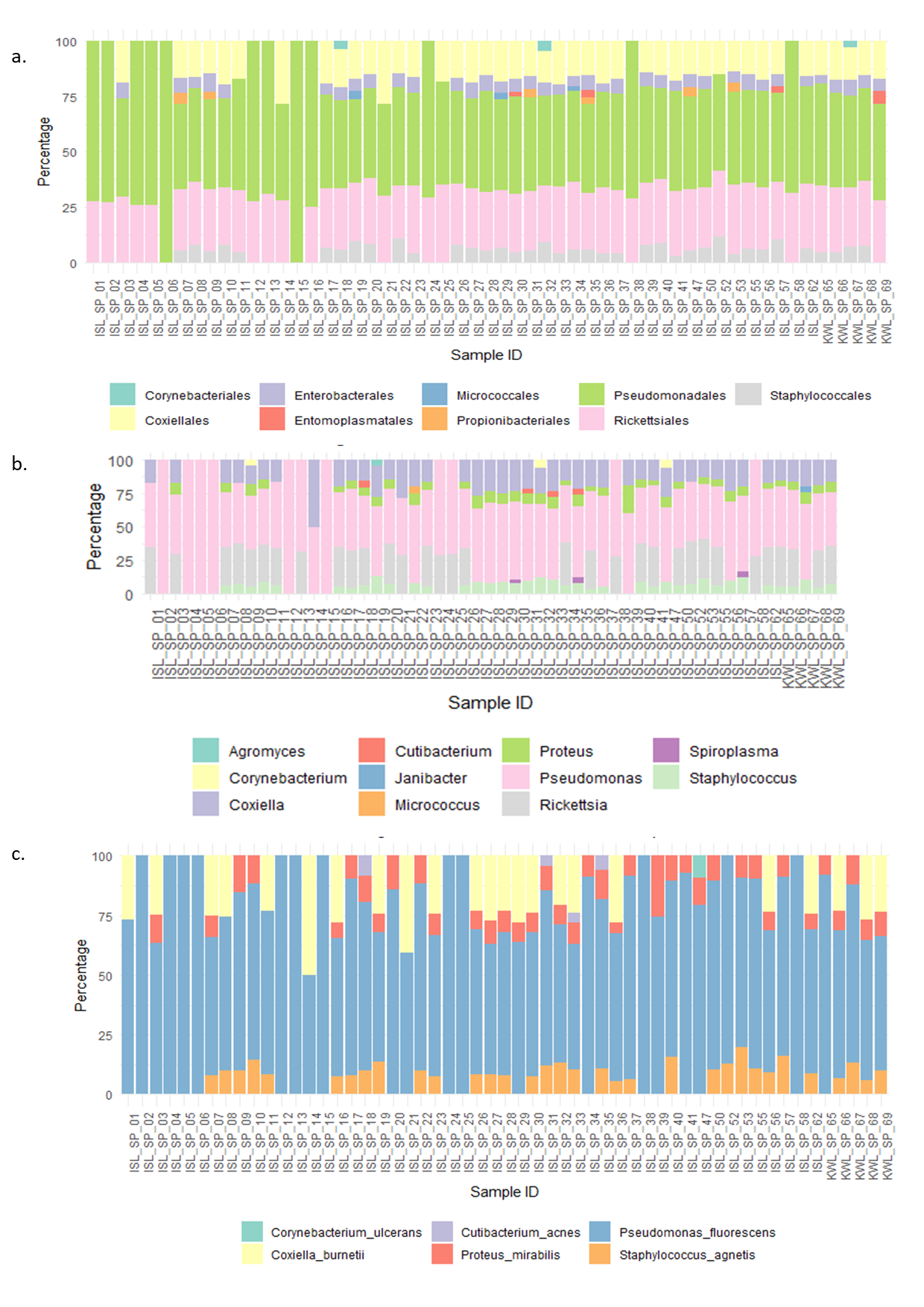

Supplement: S1 Fig — Samples with the’ ISL’ prefix are from Isiolo while those with the ’KWL’ prefix are from Kwale. (TIF) [file pone.0296597.s001.tif]
