# Supplementary material for: Detection of pathogenic bacteria in ticks from Isiolo and Kwale counties of Kenya using metagenomics
Source: PLoS One. 2024 Apr 30;19(4):e0296597. doi: 10.1371/journal.pone.0296597 (PMC11060535; doi:10.1371/journal.pone.0296597)
Supplement: S1 Table — (PDF) [file pone.0296597.s002.pdf]

| County | ISIOLO | | | | KWALE | | TOTALS | GENUS SUB-TOTALS |
|---|---|---|---|---|---|---|---|---|
| **Site** | **Market** | **Merti** | **Shambole** | **Slaughter** | **Mlalani** | **Kisima** | | |
| ***Amblyomma gemma*** | 235 | 2 | 0 | 271 | 0 | 0 | 508 | 1300 |
| *Amblyomma lepidium* | 207 | 130 | 0 | 264 | 0 | 0 | 601 | |
| *Amblyomma variegatum* | 52 | 0 | 1 | 138 | 0 | 0 | 191 | |
| *Hyalomma albiparmatum* | 0 | 0 | 0 | 1 | 5 | 0 | 6 | 376 |
| *Hyalomma dromedarii* | 13 | 3 | 0 | 10 | 0 | 0 | 26 | |
| *Hyalomma marginatum rufipes* | 99 | 3 | 0 | 210 | 0 | 0 | 312 | |
| *Hyalomma truncatum* | 32 | 0 | 0 | 0 | 0 | 0 | 32 | |
| *Rhipicephalus appendiculatus* | 319 | 107 | 0 | 698 | 0 | 0 | 1124 | 1242 |
| *Rhipicephalus boophilus decoloratus* | 3 | 1 | 0 | 0 | 26 | 15 | 45 | |
| *Rhipicephalus pulchellus* | 59 | 0 | 0 | 0 | 5 | 9 | 73 | |
| **SITE SUB-TOTALS** | 1019 | 246 | 1 | 1592 | 36 | 24 | | |
| **COUNTY TOTALS** | 2858 | | | | 60 | | **2918** | |
