# Supplementary material for: Detection of pathogenic bacteria in ticks from Isiolo and Kwale counties of Kenya using metagenomics
Source: PLoS One. 2024 Apr 30;19(4):e0296597. doi: 10.1371/journal.pone.0296597 (PMC11060535; doi:10.1371/journal.pone.0296597)
Supplement: Attachment — Submitted filename: Response to Reviewers.docx [file pone.0296597.s003.docx]

Bryson Brian Kimemia,

United States Army Medical Research Directorate-Africa (USAMRD-A),

Kenya Medical Research Institute,

P.O. Box 54840 00200 Off Raila Odinga Way, Nairobi,

Kenya.

27^th^ February 2024

Dear Dr. Shawky M Aboelhadid,

Thank you for your timely and very insightful editorial and academic review on our paper entitled “Detection of pathogenic bacteria in ticks from Isiolo and Kwale counties of Kenya using metagenomics.” We have gone through the reviews and incorporated them into the manuscript as shown in the following pages. The line numbers in the responses correspond to the line numbers in the tracked corrected manuscript.

We are thankful for your consideration in our endeavor to share our research with the scientific community and the rest of the world. We hope that this updated manuscript will meet your publication standards.

Sincerely,

Bryson Brian Kimemia

**Responses to Editorial Comments**

**Comment 1:** Please ensure that your manuscript meets PLOS ONE’s style requirements, including those for file naming. The PLOS ONE style templates can be found at

https://journals.plos.org/plosone/s/file?id=wjVg/PLOSOne_formatting_sample_main_body.pdf and

https://journals.plos.org/plosone/s/file?id=ba62/PLOSOne_formatting_sample_title_authors_affiliations.pdf

**Response 1:** The manuscript has been formatted according to PLOS ONE’s styling requirements.

**Comment 2:** In your Methods section, please provide additional information regarding the permits you obtained for the work. Please ensure you have included the full name of the authority that approved the field site access and, if no permits were required, a brief statement explaining why.

**Response 2:** Ethical approval was sought from Kenya Medical Research Institute’s (KEMRI) Scientific and Ethics Review Unit (SERU) and permission to carry out the study in the country was provided by the National Council for Science, Technology and Innovation (NACOSTI) as outlined in the Ethical Approval section.

**Comment 3:** We suggest you thoroughly copyedit your manuscript for language usage, spelling, and grammar. If you do not know anyone who can help you do this, you may wish to consider employing a professional scientific editing service.

Whilst you may use any professional scientific editing service of your choice, PLOS has partnered with both American Journal Experts (AJE) and Editage to provide discounted services to PLOS authors. Both organizations have experience helping authors meet PLOS guidelines and can provide language editing, translation, manuscript formatting, and figure formatting to ensure your manuscript meets our submission guidelines. To take advantage of our partnership with AJE, visit the AJE website (http://learn.aje.com/plos/) for a 15% discount off AJE services. To take advantage of our partnership with Editage, visit the Editage website (www.editage.com) and enter referral code PLOSEDIT for a 15% discount off Editage services.  If the PLOS editorial team finds any language issues in text that either AJE or Editage has edited, the service provider will re-edit the text for free.

Upon resubmission, please provide the following:

The name of the colleague or the details of the professional service that edited your manuscript

A copy of your manuscript showing your changes by either highlighting them or using track changes (uploaded as a *supporting information* file)

A clean copy of the edited manuscript (uploaded as the new *manuscript* file)

**Response 3:** The manuscript was reviewed, edited, and any grammatical and editorial errors corrected by the authors as evidenced by the attached tracked corrected copy of the manuscript.

**Comment 4:** Thank you for stating the following in the Acknowledgments Section of your manuscript:

“We thank Francis Ngere, Nicholas Odemba, David Oullo, Charles Waga, Richard Ochieng, Daniel Ngonga and Vitalice Opondo for their expert contribution in ticks sampling and identification.

This work was funded by the Armed Forces Health Surveillance Branch (AFHSB) and its Global Emerging Infections Surveillance (GEIS) Section, FY2022 ProMIS ID: P0116_22_KY and FY2023 ProMIS ID P0094_23_KY. The material has been reviewed by the Walter Reed Army Institute of Research. There is no objection to its presentation and/or publication. The opinions or assertions contained herein are the private views of the authors and are not to be construed as official or as reflecting the true views of the Department of the Army or the Department of Defence.”

We note that you have provided funding information that is not currently declared in your Funding Statement. However, funding information should not appear in the Acknowledgments section or other areas of your manuscript. We will only publish funding information present in the Funding Statement section of the online submission form.

Please remove any funding-related text from the manuscript and let us know how you would like to update your Funding Statement. Currently, your Funding Statement reads as follows:

“The author(s) received no specific funding for this work.”

Please include your amended statements within your cover letter; we will change the online submission form on your behalf.

**Response 4:** The funding statement was removed from the Manuscript text and the authors would like to update the Funding Statement in the submission to read “This work was funded by the Armed Forces Health Surveillance Branch (AFHSB) and its Global Emerging Infections Surveillance (GEIS) Section, FY2022 ProMIS ID: P0116_22_KY and FY2023 ProMIS ID P0094_23_KY.”

**Comment 5:** When completing the data availability statement of the submission form, you indicated that you will make your data available on acceptance. We strongly recommend all authors decide on a data sharing plan before acceptance, as the process can be lengthy and hold up publication timelines. Please note that, though access restrictions are acceptable now, your entire data will need to be made freely accessible if your manuscript is accepted for publication. This policy applies to all data except where public deposition would breach compliance with the protocol approved by your research ethics board. If you are unable to adhere to our open data policy, please kindly revise your statement to explain your reasoning and we will seek the editor’s input on an exemption. Please be assured that, once you have provided your new statement, the assessment of your exemption will not hold up the peer review process.

**Response 5:** The sequence data uploaded to NCBI was not available by the time of manuscript submission. It was successfully reviewed and can be accessed under the BioProject Accession Number **PRJNA1053293.** The sequence data has been assigned BioSample accession numbers **SAMN38855066** to **SAMN38855120**. The sequence data has been assigned **SRA** accession numbers (accession numbers - **SRS19927750, SRS19927751, SRS19927763, SRS19927772, SRS19927785, SRS19927795, SRS19927801, SRS19927802, SRS19927803, SRS19927804, SRS19927752, SRS19927753, SRS19927754, SRS19927755, SRS19927756, SRS19927757, SRS19927758, SRS19927759, SRS19927760, SRS19927762, SRS19927761, SRS19927764, SRS19927765, SRS19927767, SRS19927766, SRS19927768, SRS19927769, SRS19927770, SRS19927773, SRS19927771, SRS19927774, SRS19927775, SRS19927776, SRS19927777, SRS19927778, SRS19927779, SRS19927780, SRS19927781, SRS19927782, SRS19927784, SRS19927783, SRS19927786, SRS19927787, SRS19927789, SRS19927788, SRS19927790, SRS19927792, SRS19927791, SRS19927793, SRS19927794, SRS19927798, SRS19927799, SRS19927797, SRS19927796, SRS19927800**)

**Comment 6:** We note that Figure 1 in your submission contain [map/satellite] images which may be copyrighted. All PLOS content is published under the Creative Commons Attribution License (CC BY 4.0), which means that the manuscript, images, and Supporting Information files will be freely available online, and any third party is permitted to access, download, copy, distribute, and use these materials in any way, even commercially, with proper attribution. For these reasons, we cannot publish previously copyrighted maps or satellite images created using proprietary data, such as Google software (Google Maps, Street View, and Earth). For more information, see our copyright guidelines: http://journals.plos.org/plosone/s/licenses-and-copyright.

We require you to either (1) present written permission from the copyright holder to publish these figures specifically under the CC BY 4.0 license, or (2) remove the figures from your submission:

a. You may seek permission from the original copyright holder of Figure 1 to publish the content specifically under the CC BY 4.0 license.

We recommend that you contact the original copyright holder with the Content Permission Form (http://journals.plos.org/plosone/s/file?id=7c09/content-permission-form.pdf) and the following text:

“I request permission for the open-access journal PLOS ONE to publish XXX under the Creative Commons Attribution License (CCAL) CC BY 4.0 (http://creativecommons.org/licenses/by/4.0/). Please be aware that this license allows unrestricted use and distribution, even commercially, by third parties. Please reply and provide explicit written permission to publish XXX under a CC BY license and complete the attached form.”

Please upload the completed Content Permission Form or other proof of granted permissions as an &quot;Other&quot; file with your submission.

In the figure caption of the copyrighted figure, please include the following text: “Reprinted from [ref] under a CC BY license, with permission from [name of publisher], original copyright [original copyright year].”

b. If you are unable to obtain permission from the original copyright holder to publish these figures under the CC BY 4.0 license or if the copyright holder’s requirements are incompatible with the CC BY 4.0 license, please either i) remove the figure or ii) supply a replacement figure that complies with the CC BY 4.0 license. Please check copyright information on all replacement figures and update the figure caption with source information. If applicable, please specify in the figure caption text when a figure is similar but not identical to the original image and is therefore for illustrative purposes only.

The following resources for replacing copyrighted map figures may be helpful:

USGS National Map Viewer (public domain): http://viewer.nationalmap.gov/viewer/

The Gateway to Astronaut Photography of Earth (public domain): http://eol.jsc.nasa.gov/sseop/clickmap/

Maps at the CIA (public domain): https://www.cia.gov/library/publications/the-world-factbook/index.html and https://www.cia.gov/library/publications/cia-maps-publications/index.html

NASA Earth Observatory (public domain): http://earthobservatory.nasa.gov/

Landsat: http://landsat.visibleearth.nasa.gov/

USGS EROS (Earth Resources Observatory and Science (EROS) Center) (public domain): http://eros.usgs.gov/#

Natural Earth (public domain): http://www.naturalearthdata.com/

**Response 6:** The map images were generated using ArcGIS version 10.2.2 for Desktop (Advanced License) by Samuel Owaka using GPS Coordinates collected during the sampling process. Line 124 - 126

**Responses to Reviewer Comments**

1. **Reviewer 1**

**Comment 1:** Ticks were collected from cattle, sheep, goats, and camels, therefore, these ticks must have sucked blood. Microorganisms should be affected by the blood from different hosts. When ticks were grouped, the host they parasitized should be considered. For example, Rickettsia prowazekii are commonly asociated with fleas and body lice, and it identified in ticks may be from blood. Therefore, these limitation should be added in the disscussion.
When detecting the microorganisms, the ticks were pooled in groups of 1 to 8 individuals. Therefore, it is unreasonable to calculate the prevalence of microorganisms, and even the prevalence compared to previous studies was also unreasonable.

**Response 1:** The Minimum Infection Rate (MIR) was used to represent the minimum number of ticks infected and accounts for the pooling. Line 225 – 228.

**Comment 2:** Table 4 was not shown in the text.

**Response 2:** The table was inserted into the Manuscript and updated as Table 5. Line 396 -401.

**Comment 3:** The MS should be reviewed by a native English speaker.

**Response 3:** The manuscript was reviewed, edited, and any grammatical and editorial errors corrected by the authors as evidenced by the attached tracked corrected copy of the manuscript.

**Comment 4:** Species names: with full genus name at first mention, later with abbreviated genus name throughout the text (exception: at the beginning of a sentence). Species names and genus names should be italicized. Please check throughout the text.

**Response 4:** This was done and updated all throughout the manuscript.

**Comment 5:** “while the second was to confirm the morphological tick species identification”, hence, can the COI gene of microorganisms also be amplified with the primers provided in the MS?

**Response 5:** Yes, using these primers the Cytochrome C Oxidase subunit I gene from bacteria that obtain energy from oxidative phosphorylation can be amplified.

**Comment 6:** “COI” and “p” value should be italicized.

**Response 6:** This was done and updated all throughout the manuscript.

**Comment 7:** “Spotted Fever Group Rickettsias”, replaced by “Spotted Fever Group of Rickettsiae”.

**Response 7:** This was done. Line 74.

**Comment 8:** table 4 was not shown in text, how to deternine the prevalence?

**Response 8:** The table was inserted into the Manuscript and updated as Table 5. Line 396 -401. The Minimum Infection Rate (MIR) was used to represent the minimum number of ticks infected and accounts for the pooling. Line 225 – 228.

**Comment 9:** line 135, add as before per.

**Response 9:** This was done. Line 159.

**Comment 10:** line 290, Pseudomonadales 410, please show IQR.

**Response 10:** This was done through the removal of the statement “interquartile range” that was confusing. Line 351.

**Comment 11:** line 329, add “,” after Rickettsia slovaca.

**Response 11:** This was done. Line 407.

**Comment 12:** the section “Confirmation of Vector Identity using Cytochrome Oxidase Subunit I Sequences” should be shown in section “Tick Collection”.

**Response 12:** The section was moved to the “Tick Collection” which was renamed “Tick Collection and Identification”. Lines 232 & 266 – 271.

**Comment 13:** line 367, the endosymbiotic bacteria was not identified in this study, how to “explain why the overall bacteriome of different tick species collected from the two different regions is similar to a greater extent as indicated by the alpha diversity indices”?

**Response 13:** Endosymbiotic bacteria belong to several genera which include Rickettsia and Coxiella that were identified in this study. Using 16S rRNA data the Rickettsia genus was not speciated and the authors postulate that some of these endosymbionts may be part of the unidentified *Rickettsia* species. Line 441 – 443.

2. **Reviewer 2**

**Comment 1:** L112-113: Did the authors consider pooling the ticks based on the animal host? If yes, please include it in the “Sampling and Pooling” section.

**Response 1:** Yes, the ticks were also pooled based on the animal host. This has been updated in the “Sampling and Pooling” section. Line 133.

**Comment 2:** L139-140: The word “were” was omitted. The sentence should read ”reverse primer pair were used whereas”

**Response 2:** This has been corrected. Line 168.

**Comment 3:** L145: Remove the “min” after the “denaturation for 30 sec”.

**Response 3:** This has been corrected. Line 171.

**Comment 4:** L148: Remove the “min” after the “95^o^C denaturation for 30 sec”.

**Response 4:** This has been corrected. Line 174.

**Comment 5:** Results: For the tick collection section, the authors should consider first talking about the tick collection at the various sampled areas, the tick species identified and then zoom in to the pools.

**Response 5:** The section has been reworked to incorporate the reviewer’s recommendations. Line 232 - 249

**Comment 6:** Which of the livestock sampled were mostly infested by tick species? The authors can consider including the number of ticks collected from each livestock (cattle, sheep, goat, camel) in the results.

**Response 6:** This information has been included in the “Tick Collection and Identification” section as Table 1. Lines 249 - 250

**Comment 7:** L202-206: The authors should consider revising the statements made for clarity.

**Response 7:** The statements have been reworked to improve clarity. Line 244 – 247.

**Comment 8:** L249-252: Kindly read the sentence and make the necessary corrections.

**Response 8:** This has been corrected to show the impact of overrepresented OTUs more adequately on the tick microbiome diversity. Line 302 - 306.

**Comment 9:** Table 2 captioned “Distribution of OTUs that were classified to the bacterial species level per Tick Genera” and Table 3 “The numbers of Operational Taxonomic Units (OTUs) contributed by each tick genera that were classified (>60% confidence) to the bacterial species level” are practically the same. Remove the Table 2.

**Response 9:** Table 2 (now Table 3) was about Beta diversity measured using PERMANOVA while Table 3 (now Table 4) was about the distribution of classified reads.

**Comment 10:** L402: The authors mentioned Table 4, but no Table 4 has been presented in the results.

**Response 10:** The table was inserted into the Manuscript and updated as Table 5. Line 396 -401.

**Comment 11:** There are two different captions of Figure 1 in the manuscript. I believe the second Figure 1 mentioned in the discussion (L432-434) is Figure 5. Please confirm and make the change.

**Response 11:** Figure 1 has a different caption (Line 124 – 126) to Figure 5 (Line 384 - 386).

3. **Reviewer 3**

**Comment 1:**  Line 71: correct to “….reported spotted fever group Rickettsia in the United……..”

**Response 1:** This was corrected in the Manuscript. Line 76 - 77

**Comment 2:** Line 97: “The study was also submitted for ……………..

**Response 2:** This was amended. Line 103 – 105.

**Comment 3:** Line 108: 15 mL

**Response 3:** This was amended. Line 129.

**Comment 4:** Line 121: 1 mL

**Response 4:** This was amended. Line 145.

**Comment 5:**  Line 127: 4 m/s

**Response 5:** This was amended. Line 151.

**Comment 6:** Line 128: 1000 µL, also in line 130, correct to 200 µL.

**Response 6:** This was amended. Line 152 & 154.

**Comment 7:** Line 136: 16S rRNA

**Response 7:** This was amended. Line 160.

**Comment 8:**  Line 150: is 0.8% agarose gel not too low?

**Response 8:** This concentration of Agarose gel worked best for us without any issues.

**Comment 9:** Line 201-203: The sentence is confusing. The way it began, it sounds like the total number collected from Isiolo was 2918 which is not so. 2918 was the total number collected from both Isiolo and Kwale. The sentence needs to be revised for clarity by stating explicitly, the total overall number collected, and the total collected each from Isiolo and Kwale.

**Response 9:** This was revised in the Manuscript. Line 233 -235.

**Comment 10:** Line 205: it must be exactly and not approximately.

**Response 10:** This was amended. Line 238 – 239.

**Comment 11:** Line 207: The authors have mentioned that the collected ticks fall into 10 species in total but in line 205, it was mentioned that the 372 tick pools represented 8 ticks. This is conflicting and needs to be clarified.

**Response 11:** The first part in line 207 (now 237) referred to the number of species that were identified which was 10, while the second part in line 205 (now 245) referred to the number of individual ticks that were placed in a pool.

**Comment 12:** Line 215-217: this line of text is completely distractive considering its location. Preferably, this should come immediately after line 212.

**Response 12:** This was amended and moved. Iine 242 - 243

**Comment 13:** Line 346-347: skewed in term of what? Numbers? If so, clearly and expressly state so and avoid ambiguous terms that are meaningless without been qualified.

**Response 13:** The skew was in terms of numbers collected; this has been amended. Line 419 -420.

**Comment 14:** Line 349-350”: The use of the word “respectively” in this sentence is meaningless as it has nothing to qualify here. Take a look and revise.

**Response 14:** This was amended. Line 424

**Comment 15:** Introduction: In the current form, the introduction is too generalist without focus and the background information is too scanty that attempts to provide good background. The authors need to rejig the introduction by making it more focused.

**Response 15:** Additional information has been included in the introduction, however, the majority of research done in the country concerning tick-borne pathogens has been on viruses and veterinary pathogens. The remaining have different non-standardized reporting mechanisms which make it difficult to portray a complete picture of the situation.

**Comment 16:** Methodology: They is need for the authors to provide details of the geography of the sampling locations including details of the average temperature, humidity, and seasons. I understand that part of this information has been provided in the introduction; however, this can be removed and transferred to the methodology. Usually, the first part of the methodology deals with the description of the “study area”. In the study area, the authors should state specifically, the months and year when tick sampling was undertaken. In other words, the duration of sampling is necessary.

**Response 16:** More information has been provided in terms of collection dates, average temperature (High and Low), humidity, and season during collection. Line 110 – 117.

**Comment 17:** Discussion: Some of the results were embedded within the discussion section which is a mis normal and needs to be revised. For instance, table 2 and figure 1 was presented in between the text for the discussion. Take another look at this and address my concerns.

**Response 17:** This has been revised and all embedded results removed from the discussion.

**Comment 18:** Results: Confirmation of the vector identity should be the first part of the results after stating the total number of ticks collected across all the study locations and the number of ticks. It is abnormal to state all the pathogens detected in the various tick species before providing details of how the tick vector identity was confirmed.

**Response 18:** This has been amended and the vector confirmation results moved to the “Tick Collection and Identification” section as recommended. Line 266 – 271.

**Comment 19:** -Numerous grammatical and editorial errors that makes it difficult to grasp the meaning of some of the sentences.

**Response 19:** The manuscript was reviewed, and edited, and any grammatical and editorial errors were corrected by the authors as evidenced by the attached tracked corrected copy of the manuscript.

**Comment 20:** In the discussion, I will suggest that the authors devise a more robust way to discuss their findings. For instance, discuss the tick prevalence and distribution first, then tick-borne alpha-proteobacteria that are potentially pathogenic, zoonotic, and non-cultivable before discussing the findings from gram-negative bacteria that can be cultured on media.

**Response 20:** The discussion has been reworked to include these recommendations highlighting the distribution of collected ticks, host feeding preferences, and taxonomy of bacteria identified focusing mostly on the pathogenic ones. Non-cultivatable gram-negative bacteria such as Rickettsia and Coxiella have been discussed with comparison to prior studies in the region followed by the implication of some cultivatable extracellular bacteria such as *C. acnes* and *P. mirabilis*. Finally, the limitations sections have been amended to include those that have been brought to light by the reviewers.
