# Supplementary material for: Detection of pathogenic bacteria in ticks from Isiolo and Kwale counties of Kenya using metagenomics
Source: PLoS One. 2024 Apr 30;19(4):e0296597. doi: 10.1371/journal.pone.0296597 (PMC11060535; doi:10.1371/journal.pone.0296597)
Supplement: Attachment — Submitted filename: Response to Reviewers R2.docx [file pone.0296597.s004.docx]

Bryson Brian Kimemia,

United States Army Medical Research Directorate-Africa (USAMRD-A),

Kenya Medical Research Institute,

P.O. Box 54840 00200 Off Raila Odinga Way, Nairobi,

Kenya.

5^th^ April 2024

Dear Dr. Shawky M Aboelhadid,

Thank you for your timely and very insightful editorial and academic review on our paper entitled “Detection of pathogenic bacteria in ticks from Isiolo and Kwale counties of Kenya using metagenomics.” We have gone through the reviews from reviewer number three and have improved the rebuttal comments to the best of our knowledge. The reviewer also mentioned that the underlying data was not available, we have deposited the raw sequencing reads in NCBI SRA under accession numbers; SRS19927750, SRS19927751, SRS19927763, SRS19927772, SRS19927785, SRS19927795, SRS19927801, SRS19927802, SRS19927803, SRS19927804, SRS19927752, SRS19927753, SRS19927754, SRS19927755, SRS19927756, SRS19927757, SRS19927758, SRS19927759, SRS19927760, SRS19927762, SRS19927761, SRS19927764, SRS19927765, SRS19927767, SRS19927766, SRS19927768, SRS19927769, SRS19927770, SRS19927773, SRS19927771, SRS19927774, SRS19927775, SRS19927776, SRS19927777, SRS19927778, SRS19927779, SRS19927780, SRS19927781, SRS19927782, SRS19927784, SRS19927783, SRS19927786, SRS19927787, SRS19927789, SRS19927788, SRS19927790, SRS19927792, SRS19927791, SRS19927793, SRS19927794, SRS19927798, SRS19927799, SRS19927797, SRS19927796, SRS19927800.

We are thankful for your consideration in our endeavor to share our research with the scientific community and the rest of the world. We hope that this updated manuscript will meet your publication standards. Please do not hesitate to reach out to the corresponding author in case you need additional information.

Sincerely,

Bryson Brian Kimemia

**Responses to Reviewer Comments**

The line numbers in the responses (red) are based on the clean copy of the manuscript.

**Reviewer 3**

**Comment 1:**  Line 71: correct to “…. reported spotted fever group Rickettsia in the United……..”

**Response 1:** This was corrected in the Manuscript. Line 75

**Comment 2:** Line 97: “The study was also submitted for …………….

**Response 2:** This was amended. Line 101 – 102.

**Comment 3:** Line 108: 15 mL

**Response 3:** This was amended. Line 125.

**Comment 4:** Line 121: 1 mL

**Response 4:** This was amended. Line 136.

**Comment 5:**  Line 127: 4 m/s

**Response 5:** This was amended. Line 142.

**Comment 6:** Line 128: 1000 µL, also in line 130, correct to 200 µL.

**Response 6:** This was amended. Line 143 & 145.

**Comment 7:** Line 136: 16S rRNA

**Response 7:** This was amended. Line 154.

**Comment 8:**  Line 150: is 0.8% agarose gel not too low?

**Response 8:** The concentration of 0.8% agarose gel was the best our ladder (1 Kb) separation.

**Comment 9:** Line 201-203: The sentence is confusing. The way it began, it sounds like the total number collected from Isiolo was 2918 which is not so. 2918 was the total number collected from both Isiolo and Kwale. The sentence needs to be revised for clarity by stating explicitly, the total overall number collected, and the total collected each from Isiolo and Kwale.

**Response 9:** This was revised in the Manuscript. Line 221 -223.

**Comment 10:** Line 205: it must be exactly and not approximately.

**Response 10:** This was amended. Line 233.

**Comment 11:** Line 207: The authors have mentioned that the collected ticks fall into 10 species in total but in line 205, it was mentioned that the 372 tick pools represented 8 ticks. This is conflicting and needs to be clarified.

**Response 11:** The two lines have been amended to increase the clarity and distinction between tick collection and pooling information. Line 237 and Lines 232-233

**Comment 12:** Line 215-217: this line of text is completely distractive considering its location. Preferably, this should come immediately after line 212.

**Response 12:** This was amended and moved. Iine 230-231

**Comment 13:** Line 346-347: skewed in term of what? Numbers? If so, clearly and expressly state so and avoid ambiguous terms that are meaningless without been qualified.

**Response 13:** The skew was in terms of numbers collected; this has been amended. Line 394 -395.

**Comment 14:** Line 349-350”: The use of the word “respectively” in this sentence is meaningless as it has nothing to qualify here. Take a look and revise.

**Response 14:** The word “respectively” was removed from the highlighted section in the manuscript. Line 398.

**Comment 15:** Introduction: In the current form, the introduction is too generalist without focus and the background information is too scanty that attempts to provide good background. The authors need to rejig the introduction by making it more focused.

**Response 15:** The authors have reviewed the introduction and have included additional information to make it more focused. Lines 70 – 73 & Lines 80 - 90

**Comment 16:** Methodology: They is need for the authors to provide details of the geography of the sampling locations including details of the average temperature, humidity, and seasons. I understand that part of this information has been provided in the introduction; however, this can be removed and transferred to the methodology. Usually, the first part of the methodology deals with the description of the “study area”. In the study area, the authors should state specifically, the months and years when tick sampling was undertaken. In other words, the duration of sampling is necessary.

**Response 16:** More information has been provided in terms of collection dates, average temperature (High and Low), humidity, and season during collection. Line 112 – 119.

**Comment 17:** Discussion: Some of the results were embedded within the discussion section which is a mis normal and needs to be revised. For instance, table 2 and figure 1 were presented in between the text for the discussion. Take another look at this and address my concerns.

**Response 17:** This has been revised and all embedded results removed from the discussion.

**Comment 18:** Results: Confirmation of the vector identity should be the first part of the results after stating the total number of ticks collected across all the study locations and the number of ticks. It is abnormal to state all the pathogens detected in the various tick species before providing details of how the tick vector identity was confirmed.

**Response 18:** This has been amended and the vector confirmation results moved to the “Tick Collection and Identification” section as recommended. Line 246 – 251.

**Comment 19:** -Numerous grammatical and editorial errors that make it difficult to grasp the meaning of some of the sentences.

**Response 19:** The manuscript was reviewed, and edited, and any grammatical and editorial errors were corrected by the authors as evidenced by the attached tracked corrected copy of the manuscript.

**Comment 20:** In the discussion, I will suggest that the authors devise a more robust way to discuss their findings. For instance, discuss the tick prevalence and distribution first, then tick-borne alpha-proteobacteria that are potentially pathogenic, zoonotic, and non-cultivable before discussing the findings from gram-negative bacteria that can be cultured on media.

**Response 20:** The discussion has been reworked to include these recommendations highlighting the distribution of collected ticks, host feeding preferences, and taxonomy of bacteria identified focusing mostly on the pathogenic ones. Non-cultivatable gram-negative bacteria such as Rickettsia and Coxiella have been discussed with comparison to prior studies in the region followed by the implication of some cultivatable extracellular bacteria such as *C. acnes* and *P. mirabilis*. Finally, the limitations sections have been amended to include those that have been brought to light by the reviewers.
